# Enhanced immunoprecipitation techniques for the identification of RNA-binding protein partners: IGF2BP1 interactions in mammary epithelial cells

**DOI:** 10.1016/j.jbc.2022.101649

**Published:** 2022-01-29

**Authors:** Saja A. Fakhraldeen, Scott M. Berry, David J. Beebe, Avtar Roopra, Celia M. Bisbach, Vladimir S. Spiegelman, Natalie M. Niemi, Caroline M. Alexander

**Affiliations:** 1 McArdle Laboratory for Cancer Research, University of Wisconsin-Madison, Madison, Wisconsin, USA; 2 Department of Biomedical Engineering, University of Wisconsin-Madison, Madison, Wisconsin, USA; 3 Department of Neuroscience, University of Wisconsin-Madison, Madison, Wisconsin, USA; 4 Division of Pediatric Hematology/Oncology, Department of Pediatrics, Pennsylvania State University College of Medicine, Hershey, Pennsylvania, USA; 5 Department of Biochemistry & Molecular Biophysics, Washington University in St Louis

**Keywords:** RNA-binding protein, RNA–protein interaction, post-transcriptional regulation, breast cancer, cancer biology, cDNA, complementary DNA, EP, epithelial, ESP, Exclusion-Based Sample Preparation, GPX, glutathione peroxidase, GST, glutathione-*S*-transferase, HRP, horseradish peroxidase, IFAST, Immiscible Filtration Assisted by Surface Tension, IgG, immunoglobulin G, MEF, mouse embryonic fibroblast, MRPL, mitochondrial ribosomal protein large subunit, MRPS, mitochondrial ribosomal protein small subunit, PAR–CLIP, photoactivatable ribonucleoside–enhanced crosslinking and immunoprecipitation, PMP, paramagnetic particle, qRT–PCR, quantitative RT–PCR, RBP, RNA-binding protein, RIP, RNA immunoprecipitation, RRID, research resource identifier, SLIDE, Sliding Lid for Immobilized Droplet Extraction, STRING, Search Tool for the Retrieval of Interacting Genes/Proteins, IMP-1: VICKZ1, Vg1 RBP/Vera, IMP-1,2,3, CRD–BP, KOC, ZBP-1 family member 1, CRD-BP, coding region determinant-binding protein, ZBP1, zipcode-binding protein-1, IGF2BP1, insulin-like growth factor 2 mRNA-binding protein 1

## Abstract

RNA-binding proteins (RBPs) regulate the expression of large cohorts of RNA species to produce programmatic changes in cellular phenotypes. To describe the function of RBPs within a cell, it is key to identify their mRNA-binding partners. This is often done by crosslinking nucleic acids to RBPs, followed by chemical release of the nucleic acid fragments for analysis. However, this methodology is lengthy, which involves complex processing with attendant sample losses, thus large amounts of starting materials and prone to artifacts. To evaluate potential alternative technologies, we tested “exclusion-based” purification of immunoprecipitates (IFAST or SLIDE) and report here that these methods can efficiently, rapidly, and specifically isolate RBP–RNA complexes. The analysis requires less than 1% of the starting material required for techniques that include crosslinking. Depending on the antibody used, 50% to 100% starting protein can be retrieved, facilitating the assay of endogenous levels of RBPs; the isolated ribonucleoproteins are subsequently analyzed using standard techniques, to provide a comprehensive portrait of RBP complexes. Using exclusion-based techniques, we show that the mRNA-binding partners for RBP IGF2BP1 in cultured mammary epithelial cells are enriched in mRNAs important for detoxifying superoxides (specifically glutathione peroxidase [GPX]-1 and GPX-2) and mRNAs encoding mitochondrial proteins. We show that these interactions are functionally significant, as loss of function of IGF2BP1 leads to destabilization of GPX mRNAs and reduces mitochondrial membrane potential and oxygen consumption. We speculate that this underlies a consistent requirement for IGF2BP1 for the expression of clonogenic activity *in vitro*.

RNA-binding proteins (RBPs) are critical post-transcriptional regulators of gene expression in normal and pathological cellular contexts (1). At least 1542 RBPs govern RNA metabolism at myriad stages of splicing, export, storage, transport, and translation (2). Often, RBPs bind select RNA species to modulate their expression, localization, and/or stability, occasionally *via* highly specific and conserved sequence motifs. However, more typically, RBPs bind RNA species *via* short and degenerate sequences that are not easy to recognize prospectively (1).

Aberrant RBP activity is responsible for such important phenotypes as fragile X syndrome (*via* the RBP fragile X mental retardation protein) (3), and splicing reactions of cancer-associated tumor drivers, such as androgen receptor (*via* the RBP DEAD-box helicase 3 X-linked) (4). It is therefore important to define the cohort of mRNA-binding partners that are bound by each specific RBP, since these partners are likely to be affected by altered RBP expression or activity. The cohort of mRNA species bound by a given RBP can be highly cell type specific for reasons that are not yet understood. RBPs sometimes stabilize mRNA species; this is deduced from the demonstration of a direct binding interaction, together with decreased abundance upon RBP knockdown/knockout (1, 5). However, other regulatory activities that do not result in altered RNA abundance are much more difficult to identify, for example, regulation of RNA localization and delivery of target proteins to specific subcellular structures (6). Some studies have selected specific mRNAs with obvious roles to show that RBP–mRNA interactions regulate function; for example, Conway *et al.* (7) demonstrated adhesion defects after disruption of an insulin-like growth factor 2 mRNA-binding protein 1 (IGF2BP1)–integrin subunit beta 5 mRNA in embryonic stem cells.

Most studies rely upon UV-induced crosslinking coupled with immunoprecipitation techniques to define mRNA-binding partners for RBPs. By exploiting the unique chemical reactivity of RNA for protein, irreversible crosslinks can be formed between RNA and protein moieties that lie in close proximity (1). This technique was widely adopted after concerns were raised about the potential for switching of RBP-binding partners during incubations (8). However, it is not trivial to reverse these crosslinks sufficiently to release and identify the bound mRNA species, and the yield of input RBP that emerges after the extensive processing reactions is substantially less than 1% of total (9). Various versions of photoactivatable ribonucleoside–enhanced crosslinking and immunoprecipitation (PAR–CLIP) protocols have been described and applied to the analysis of RBPs; the strengths and weaknesses of each have been reviewed (5, 9, 10, 11, 12). In general, the limitations of crosslinking protocols fall into various classes: the loss of unstable RNA species during long processing procedures, loss of mRNAs with indirect or low-affinity interactions during washing of immunoprecipitates, artifacts created by crosslinking and extensive derivatization processes, and the requirement for an impractically high starting numbers of labeled cultured cells overexpressing the RBP of interest. Indeed, in many key cell types, RBP interactions cannot be studied because of the requirement for up to 1 g of starting protein lysate. Here, we have applied two Exclusion-Based Sample Preparation (ESP) technologies to identify mRNA-binding partners for an exemplar RBP; the simple and expedited processing that is required for the techniques used in this article *versus* those required for PAR–CLIP is summarized in Figure 1.

**Figure 1:**
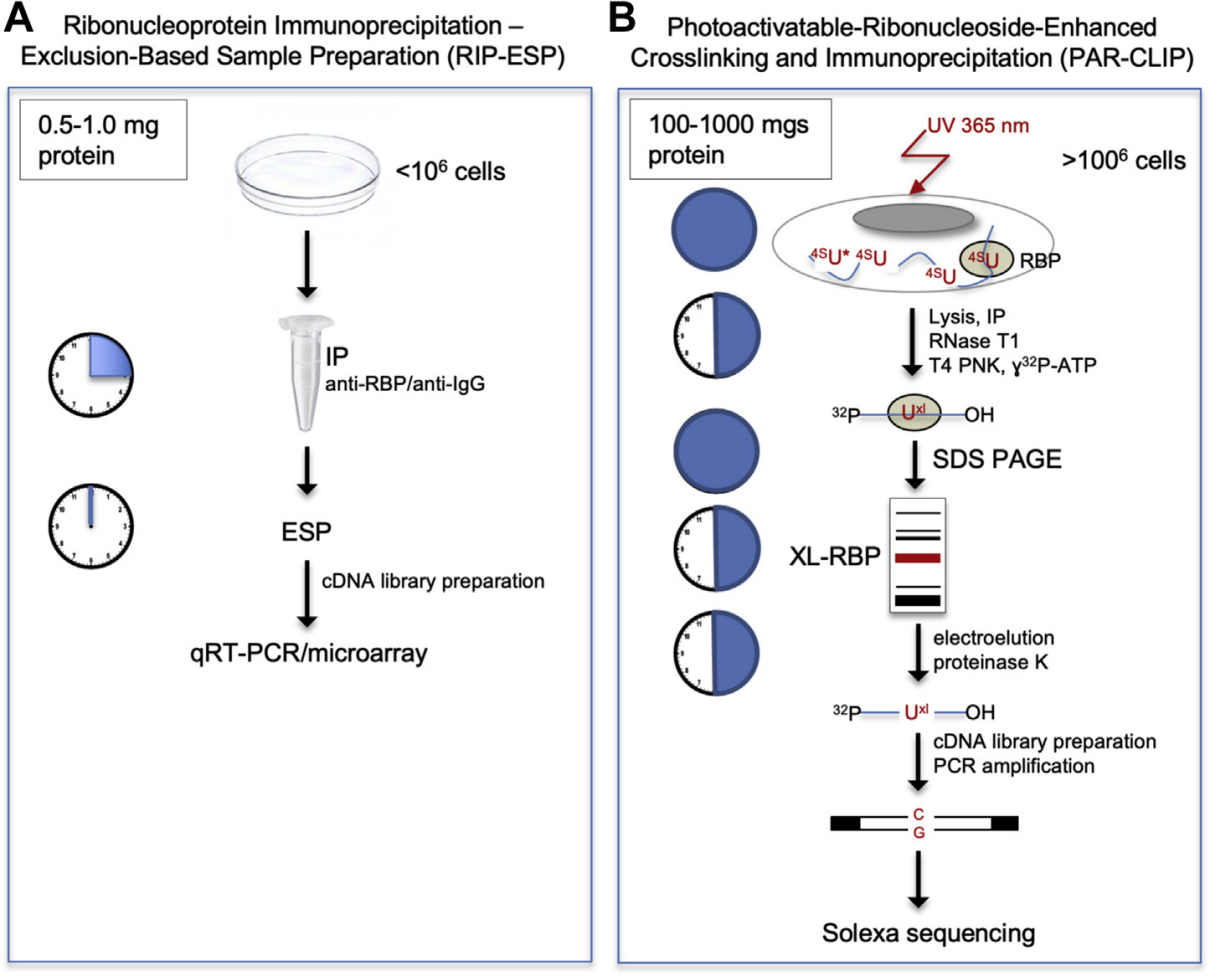
**Comparison of ESP and PAR–CLIP technologies.** Schematics of the workflow for ESP methodology (*A*) compared with PAR–CLIP (*B*), as performed by Hafner *et al.* (39), to illustrate one of the incentives for performing this study. Specifically, the timeline and handling required for both procedures is shown on clock faces, together with the relative amounts of starting cell lysate required, and the complexity of the PAR–CLIP protocol. Typically, RBPs are overexpressed for CLIP protocols. ∗ indicates the use of nucleoside substitution, which can induce a nucleolar stress response and result in cytotoxicity (81, 82). ESP, Exclusion-Based Sample Preparation; PAR–CLIP, photoactivatable ribonucleoside–enhanced crosslinking and immunoprecipitation; RNA-binding protein.

The focus of this study is the RBP IGF2BP1 (IGF2 mRNA-binding protein-1 [IMP-1]), which is known by several other names, depending on the activity ascribed by several independent investigators (13, 14, 15, 16). Thus, coding region determinant-binding protein was originally defined as a regulator of stability of MYC mRNA and a modulator of βTrCP, a ubiquitin ligase regulating Wnt signaling (17, 18); other names reflect other functions, including the regulated delivery of β-actin to cell lamellae (zipcode-binding protein-1, ZBP1; the complete list of alternate names [IGF2BP1 or IMP-1; CRD-BP; and VICKZ1] is provided in the Abbreviations section).

Although often described as showing an oncofetal expression pattern (expressed in embryo, not adult tissues, and re-expressed by tumors), we demonstrated significant and approximately similar expression in breast tumor and nontumor cells. The predominant isomer expressed in both conditions was an N-truncated variant initiating from an internal promoter, containing all the mRNA-binding KH domains (19). Expression levels were approximately 100× lower than for embryonic cells (either mouse embryonic fibroblasts [MEFs] or 293T cells). Nonetheless, this protein regulated clonogenic growth *in vitro* (19). Indeed, IMP-1 has been shown to be required for clonogenic activity in many tumor cell types, suggesting that it enables some fundamental property required for clonogenic growth (20). Other tumor-associated activities have also been linked to IMP-1 expression (21, 22, 23, 24, 25).

Target mRNAs bind the KH repeat domains of IMP-1 *via* combinatorial interactions through a looped tertiary structure with short consensus sequences. This complex interaction makes IMP-1 mRNA-binding partners difficult to predict *a priori* (26, 27). Since endogenous IMP-1 is expressed at such low levels in breast epithelial (EP) cells, the isolation of binding partners has been a challenge. Therefore, we turned to ESP preparation technologies because of their inherent sensitivity, speed, parallel processing capacity, and potential for multiple endpoint assays (28). Briefly, these techniques allow for the gentle and reliable extraction of analyte-bound paramagnetic particles (PMPs) by magnetically immobilizing and removing PMPs from incubation and wash buffers, thereby minimizing the time spent in large wash buffer volumes. By leveraging the surface tension of fluids, samples can be purified within seconds, prohibiting dissociation (and reassociation with noncognate targets), which typically occurs during typical RNA immunoprecipitation (RIP) protocols. We previously showed that low-affinity interactions dissociated after 10 min of wash incubation (29). The two techniques tested here evaluate two versions of this hydrophilic–hydrophobic ESP patterning, one oil based (Immiscible Filtration Assisted by Surface Tension [IFAST]) and the other air based (Sliding Lid for Immobilized Droplet Extractions [SLIDE]).

The mRNA-binding partners identified by this analysis include a group of mRNAs encoding proteins destined for mitochondria and several mRNAs encoding proteins involved in glutathione metabolism such as the selenoprotein glutathione peroxidases GPX-1 and GPX-2 (important for the detoxification of superoxides (30)). We found that mitochondrial function and GPX-2 mRNA stability required IMP-1 in breast EP cells, confirming previously described regulatory binding reactions of mRNAs with related functions in other cell types (31, 32). Given the high sensitivity and accuracy of these ESP techniques, we propose that this technology will be a useful approach to dissecting RBP function in general, either alone or as a complement to techniques that provide accurate binding site predictions derived from crosslinking (CLIP) studies.

## Results

### Use of ESP devices

#### IFAST

**Figure 2:**
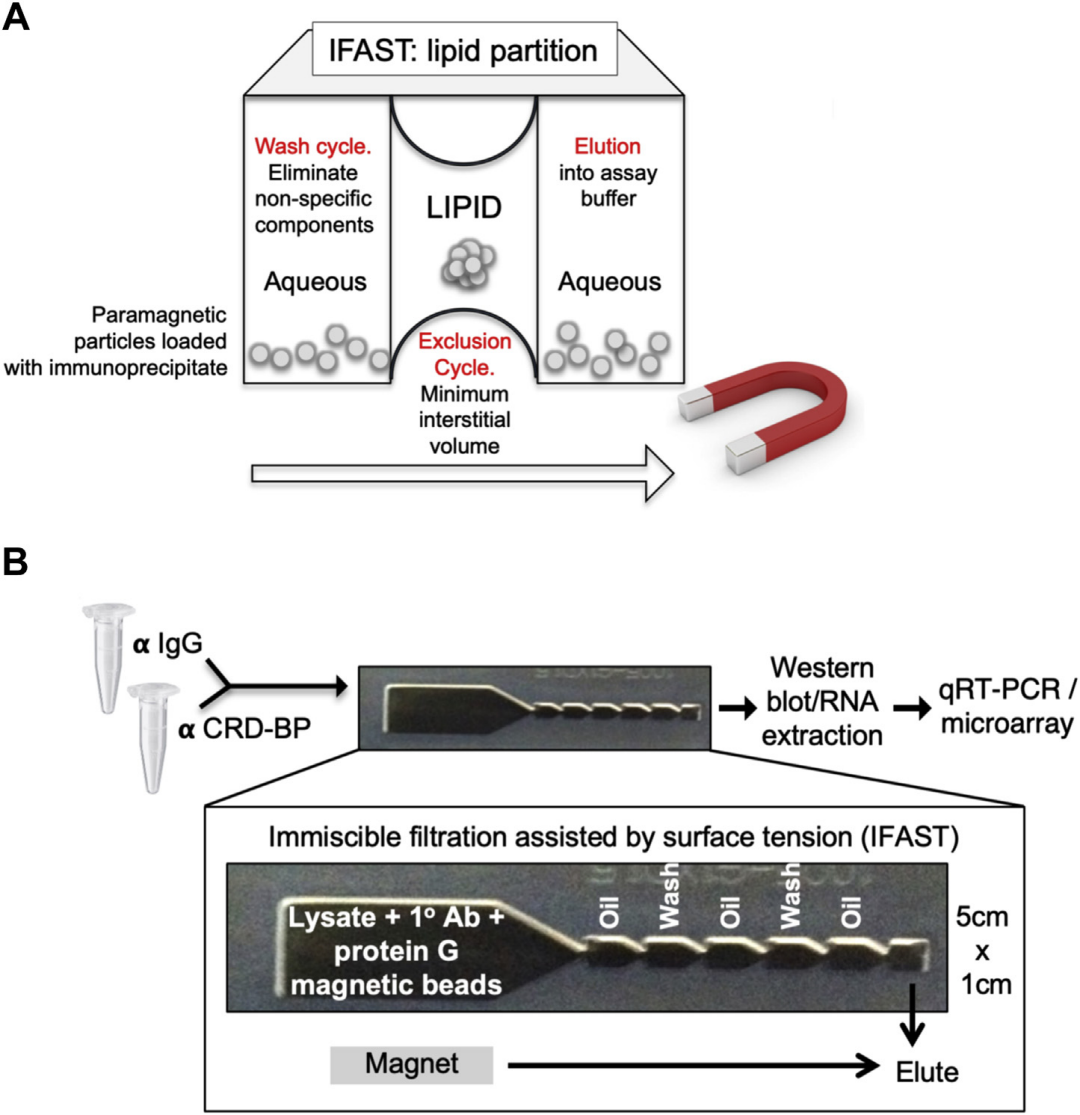
**Overview of ESP-based Immiscible Filtration Assisted by Surface Tension (IFAST).***A*, schematic of IFAST–RIP technique; the purification (or “exclusion”) phase of the ribonucleoprotein immunoprecipitation process is provided by pulling magnetic beads loaded with immunoprecipitate through a lipid barrier located between aqueous wells. *B*, an overview of the IFAST protocol, showing the configuration and dimensions of the device. ESP, Exclusion-Based Sample Preparation; RIP, RNA immunoprecipitation.

IFAST devices are fabricated from polypropylene *via* injection compression molding (DTE Research and Design, LLC) and consist of linearly aligned wells (volume of 5–15 μl) connected by trapezoidal microfluidic conduits (Fig. 2). These wells are flanked by a larger input well (up to 200 μl volume) on one end and an output well of designer-specified volume (5–10 μl) on the other. The preincubated cell lysate–antibody–PMP (prepared as described in the Experimental procedures section) mixture is transferred to the input well, and PMP-bound biomolecular complexes are purified by a magnet-based pull through the intermediate wells, which consist of alternating solutions of oil (Fluorinert FC-40 oil; Sigma–Aldrich), and aqueous wash phases, to the output well. Note that this requires no pipetting or additional handling beyond the initial loading of the device and takes an average of 20 to 30 s. The utility of this device for identifying valid biological interactions (including weak interactions), for streamlining multiplexed assays of analytes, and for the detection of viral RNAs for clinical diagnostics has been previously demonstrated (29, 33, 34, 35, 36).

#### SLIDE

**Figure 3:**
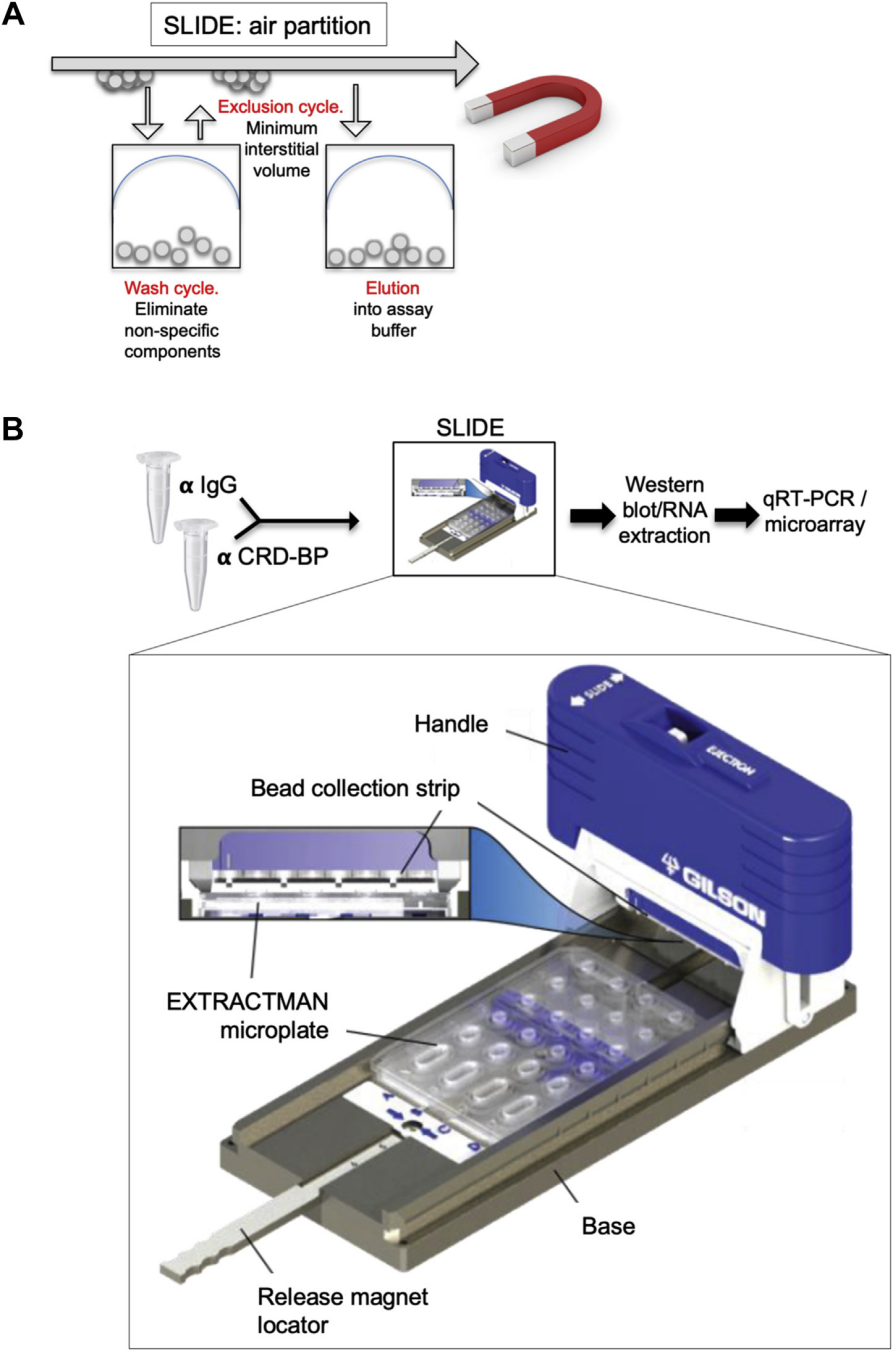
**Overview of ESP-based Sliding Lid for Immobilized Droplet Extraction (SLIDE).***A*, schematic of SLIDE technique using EXTRACTMAN device (Gilson, Inc). The exclusion phase operates by repeated cycles of lifting of magnetic beads loaded with immunoprecipitate out of the wash solution. The extracted drops are held against a hydrophobic surface with minimum aqueous volume and surface tension before re-elution in a fresh aqueous solution. *B*, an overview of the SLIDE protocol, showing the configuration and dimensions of this specific device. ESP, Exclusion-Based Sample Preparation.

To avoid the use of oil-based exclusion, we used a SLIDE device, which depends instead on air-based exclusion (37). This has the advantage of eliminating oil from the purification process and the pull-through lysate. The SLIDE device consists of a handle and a base, each with movable magnets within them (37) (commercial name is EXTRACTMAN from Gilson). A polypropylene well plate (provided by Gilson, Inc) is loaded with samples containing PMPs, wash buffer, and elution buffer. By sliding the SLIDE handle across the base, PMPs are rapidly and efficiently transferred between reagents in series. Importantly, PMPs are collected on a disposable PMP collection strip, which comprises highly polished uncharged polypropylene. Thus, this hydrophobic PMP collection minimizes carryover of aqueous material as the SLIDE handle moves between reagents. In RIP experiments, the input wells of this device are loaded with cell lysate, and the RBP complex–bound PMP beads are moved through adjacent wells containing wash buffer as described previously (Fig. 3); total time for exclusion purification is approximately 20 s.

By passing the PMPs carrying immunoprecipitation complexes through oil (IFAST) or air (SLIDE) by attraction to a magnet, the aqueous dead volumes are minimized, reducing the time and handling required to dilute out associated fluids (*i.e.*, to wash immunoprecipitates). The internal aqueous volume of PMPs is approximately 115 nl for each 5 μl volume of beads. We optimized the protocol for this specific buffer composition, given that the residual surface volume determines surface tension (increased by higher salt and decreased by detergent). Samples were processed simultaneously for up to four immuno-PMP lysates (directly in parallel), whereas samples were processed individually in the IFAST devices.

### Efficiency of purification of the RBP, IMP-1

**Figure 4:**
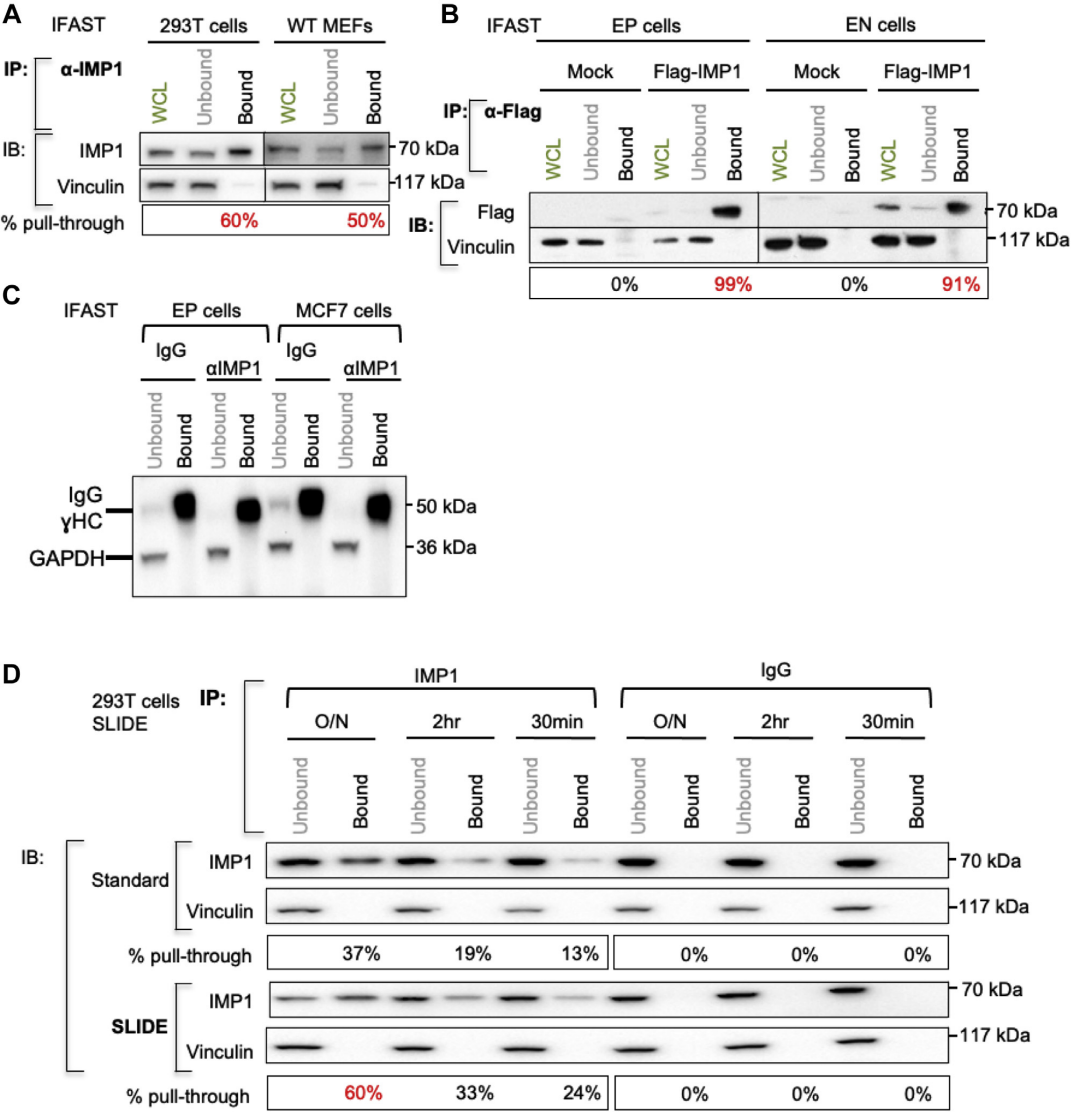
**Efficiency of pull through of IMP-1 protein using IFAST.***A*, demonstration of efficiency of pull through of endogenous IMP-1. Two cell types with high levels of endogenous IMP-1 (293T and MEF) were lysed, incubated with anti-IMP-1 primary antibody and protein G paramagnetic particles (PMPs), and RIP fractions purified using IFAST. A known amount (typically 20%) of total immunoprecipitate pulled through was analyzed by Western blotting (bound) and compared with the input remaining (unbound), with whole cell lysate (WCL; before pull through) shown for comparison. Vinculin is used to evaluate specificity of the immunoprecipitation. The fraction of IMP-1 pulled through is shown (in percentage). Results shown are representative of three experiments. *B*, comparison of efficiency of pull through of tagged IMP-1. Mouse mammary cell lines, EP and EN cells, were transfected with FLAG-tagged IMP-1 (or empty vector, mock). About 48 h later, lysates were purified using IFAST. Results shown are representative of three experiments. *C*, evaluation of efficiency of pull through of antibody–PMP particles using IFAST. For the cell lysates indicated, the amount of IgG remaining in the unbound fraction was assessed by Western blotting with conformation-sensitive anti-IgG antibody. *D*, evaluation of efficiency of immunoprecipitation with time. 293T cell lysates were incubated with anti-IMP-1 antibody or an IgG control and PMPs for varying lengths of time (overnight [O/N], 2 h, or 30 min) before purification either by standard or by SLIDE-based RIP. Pull-through efficiency was evaluated by Western blotting (n = 2). EP, epithelial; IFAST, Immiscible Filtration Assisted by Surface Tension; IgG, immunoglobulin G; IMP-1, insulin-like growth factor 2 mRNA-binding protein-1; MEF, mouse embryonic fibroblast; PMP, paramagnetic particle; RIP, RNA immunoprecipitation; SLIDE, Sliding Lid for Immobilized Droplet Extractions.

We tested the efficiency of the recovery of endogenous IMP-1 protein by immunoprecipitation using IFAST, first for two cell types, 293T human embryonic kidney cells and MEFs, and then for a tagged IMP-1 protein (applying a different antibody, anti-FLAG) expressed in cultured mouse mammary EP cells (Fig. 4, *A* and *B*) (38, 39). Using the anti-IMP-1 antibody to purify endogenous IMP-1 from 293T cells and MEFs, the yield of purified protein was 50% to 60% of total input; for the high-affinity anti-FLAG antibody, yield was even higher and losses were insignificant. Vinculin was used as an indicator of nonspecific protein adsorption and was not detectable. We also tested the efficiency of the affinity purification by assaying residual antibody in the unbound fraction and found almost no losses for the immunocomplexes during extraction from the cell lysates (Fig. 4*C*).

We next evaluated the efficiency of recovery when shorter times were allowed for immune complexation. Maximal recovery was found for overnight incubation, but significant recovery was obtained using only 30 min of binding (24% for 30 min compared with 60% recovery for overnight complexation) (Fig. 4*D*). For unstable RNAs or rapidly reassociating species, these short preincubation times could be particularly important.

### Efficiency of immunoprecipitation of RNA with endogenous IMP-1 protein

**Figure 5:**
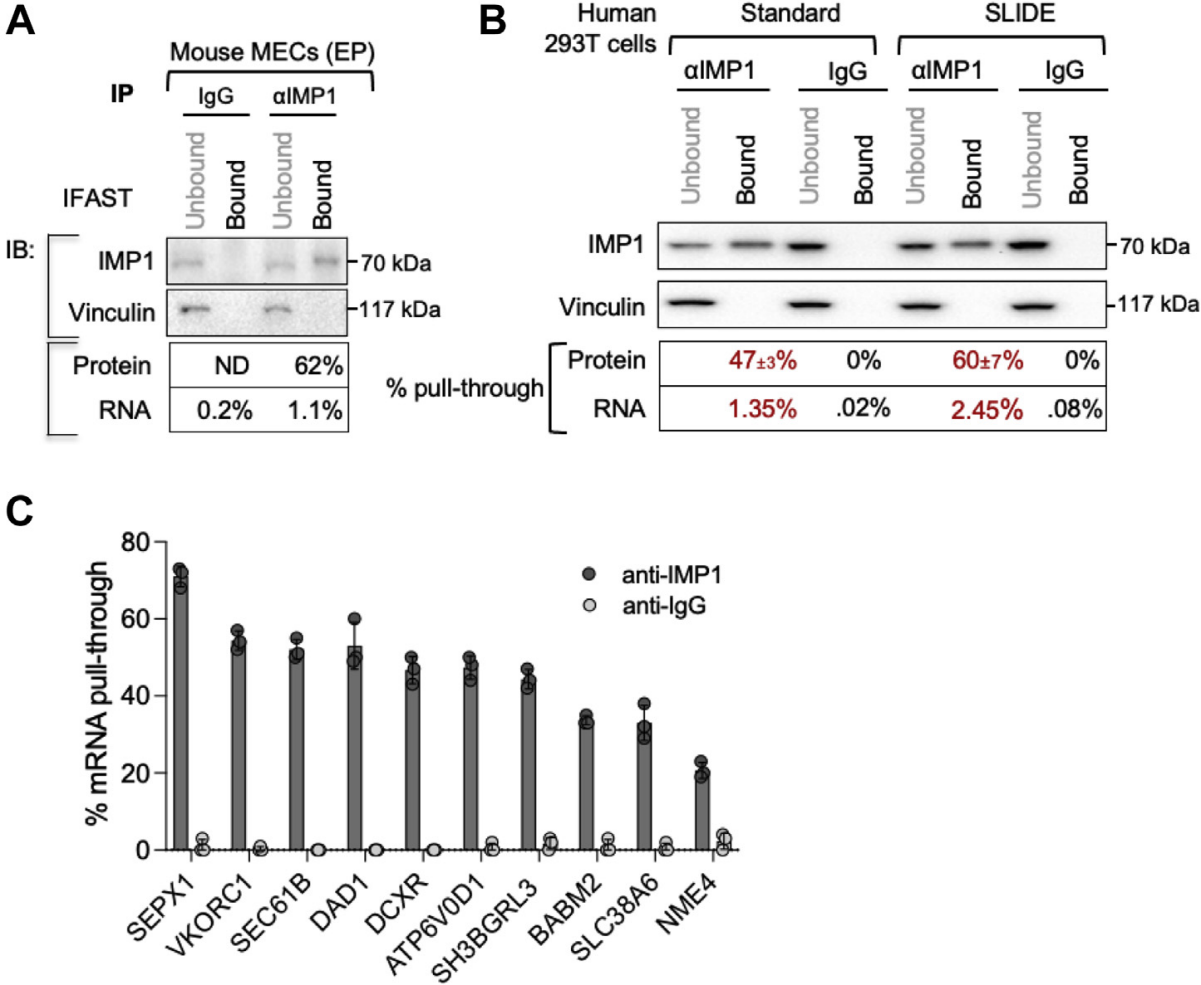
**Immunoprecipitation of RNA in RIP IMP-1 complexes isolated by ESP methods.***A*, determination of amount of RNA in IFAST–RIP. Mouse mammary epithelial (EP) cell lysates were incubated with anti-IMP-1 antibody or an IgG control, and immunoprecipitates were purified using IFAST–RIP. The protein component of the immunoprecipitate was analyzed by Western blotting as in Figure 4. RNA was purified, and the amount of RNA in each fraction was determined (n = 3). *B*, side-by-side comparison of RNA purification by standard and SLIDE–RIP. 293T cell lysates were incubated with anti-IMP-1 1° Ab or an IgG control, and lysate + antibody mixtures were purified using either standard or SLIDE-based RIP. The efficiency of pull through of IMP-1 (n = 3) and associated RNAs (n = 2) is shown. *C*, validation of SLIDE-enriched mRNA partners. Selected RNA species identified in FLAG-tagged IMP-1-associated RNP particles by Jonson *et al.* (38) were evaluated by qPCR of SLIDE-enriched RIP fractions of endogenous IMP-1 from 293T cells (n = 3). Ab, antibody; ESP, Exclusion-Based Sample Preparation; IFAST, Immiscible Filtration Assisted by Surface Tension; IgG, immunoglobulin G; IMP-1, insulin-like growth factor 2 mRNA-binding protein-1; qPCR, quantitative PCR; RIP, RNA immunoprecipitation; SLIDE, Sliding Lid for Immobilized Droplet Extractions.

To evaluate the efficiency of recovery for cells with low endogenous levels of IMP-1, we tested a nontumorigenic mouse mammary EP cell strain, EP cells (40). Although IMP-1 is typically 100× less expressed in cells derived from adults compared with fetal cells, IMP-1 is still functionally important, at least for the expression of clonogenicity *in vitro* (19). We showed that the efficiency of pull through of IMP-1 by IFAST from mouse mammary EP cells was approximately the same as for cell lines with higher endogenous levels of IMP-1 (shown in Fig. 4); here measured at 62% by Western blotting (Fig. 5*A*).

Using the IFAST protocol, fivefold more RNA was pulled through with the IMP-1 immunocomplexes than with the control (immunoglobulin G [IgG])-bound PMPs (1.1% compared with 0.2% for anti-IgG) (Fig. 5*A*). A “standard” immunoprecipitation protocol without crosslinking was compared with IFAST-purified RIP complexes; in other words, we used typical serial pipetting operations to conduct sequential and manual washes of each immunoprecipitate-bound PMP sample in individual Eppendorf tubes. We found broadly similar efficiency for recovery of both RBP and the total associated RNA (Fig. 5*B*).

We also tested whether the RNAs pulled through by this enhanced immunoprecipitation protocol included mRNA-binding partners previously characterized as IMP-1-binding partners in 293T cells (38). All 10 mRNA species surveyed were significantly pulled through by SLIDE–RIP (Fig. 5*C*).

### Analysis of the IMP-1 mRNA complexes from mouse mammary EP cells

Ranked gene lists of bound and unbound mRNA fractions were compared for IMP-1-associated or IgG-associated IFAST-purified RIP fractions to identify species that showed a significant change in rank listing (*p* < 0.01). The IMP-1 gene list included 1343 genes, of which 443 (approximately 35%) overlapped with the gene list from IgG control fractions (1170 genes). These “sticky mRNAs” were subtracted from the total to generate a list of 900 potentially specific mRNAs in IMP-1-associated complexes.

**Figure 6:**
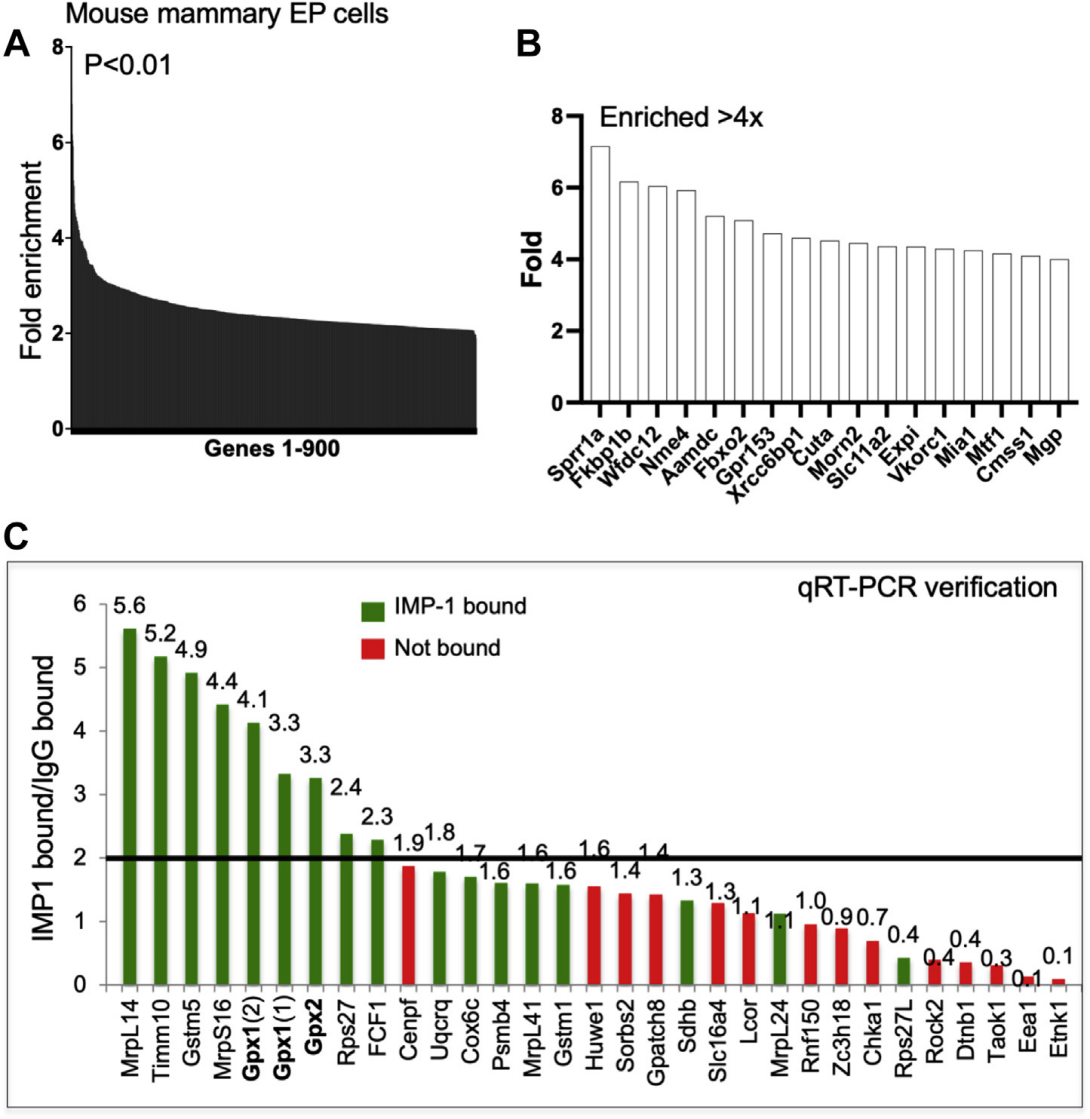
**Analysis of RNA-binding partners for IMP-1 in mouse mammary epithelial (EP) cells.***A*, general enrichment of bound mRNAs. The relative fold enrichment of 900 genes specifically enriched in the anti-IMP-1 complexes (*p* < 0.01) purified by IFAST–RIP from EP cells. *B*, most enriched mRNA species. The mRNA species greater than four times enriched are shown in detail in *A*. *C*, confirmation of mRNAs pulled through in functional groupings. Functional groupings are shown diagrammatically in Figure 7. To confirm enrichment shown from array analysis, a subset of associated mRNAs were tested by qRT–PCR analysis. For this study, a set of 31 mRNAs comprising 17 specifically and significantly enriched in the IMP-1-bound fraction (*green*) were compared with 14 mRNAs specifically excluded from the IMP-1-bound fraction (*red*). A potential thresholding line is drawn that excludes 93% of mRNAs not enriched by array analysis and includes 64% of mRNAs designated as enriched. IFAST, Immiscible Filtration Assisted by Surface Tension; IMP-1, insulin-like growth factor 2 mRNA-binding protein-1; qRT, quantitative RT; RIP, RNA immunoprecipitation.

The fold enrichment of these 900 mRNA species (all greater than twofold) is illustrated in Figure 6*A*, and the mRNAs most highly enriched are shown in Figure 6*B* (greater than fourfold). To verify the array analysis of RIP fractions, we selected >30 mRNA species for evaluation by quantitative RT–PCR (qRT–PCR), including enriched and excluded mRNAs (Fig. 6*C*). For the purpose of illustration, we set a threshold on this confirmation assay; this threshold excludes 93% of mRNAs not enriched by array analysis and also increases the stringency of inclusion in the specifically enriched fraction.

**Figure 7:**
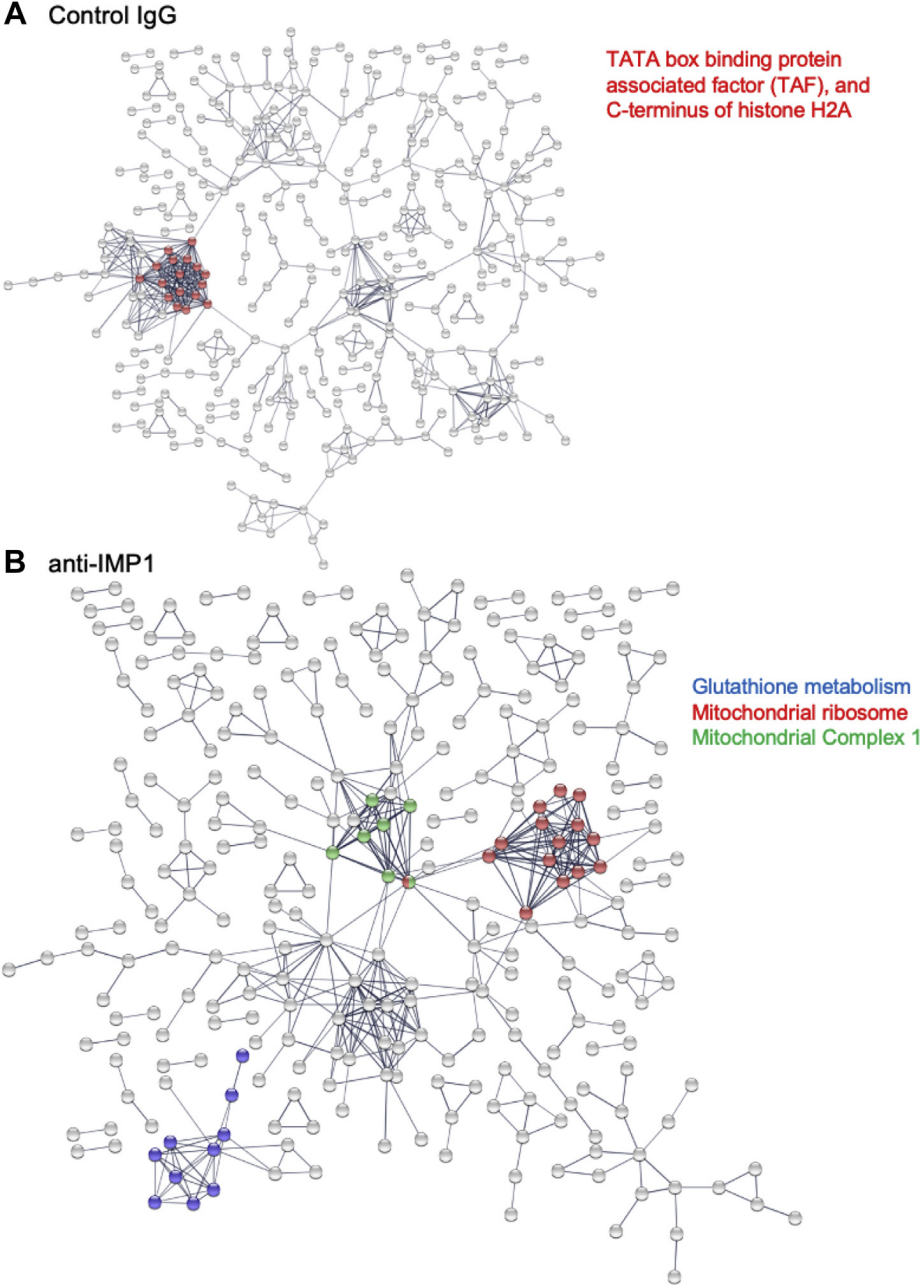
**Functional grouping of mRNAs in IMP-1 complexes from mouse mammary epithelial (EP) cells.***A*, the mRNAs in control IgG complexes were subjected to STRING analysis, which found no significant enrichment groups (defined as FDR > 0.05) except for a (relatively unique) group of histone mRNAs, and intriguingly, the mRNA for IMP-1 itself. *B*, the mRNAs in IMP-1-associated complexes showed functional enrichment for two groups of genes, glutathione metabolism genes (including GPX-2) and mRNAs for proteins targeted to mitochondria, important for mitochondrial ribosome function and the assembly of complex 1 in the electron transport chain. FDR, false discovery rate; GPX-2, glutathione peroxidase; IgG, immunoglobulin G; IMP-1, insulin-like growth factor 2 mRNA-binding protein-1; STRING, Search Tool for the Retrieval of Interacting Genes/Proteins.

To test whether this group of genes includes mRNAs significantly associated with specific cellular processes, we analyzed the group of 900 genes by Search Tool for the Retrieval of Interacting Genes/Proteins analysis (Fig. 7). We found significant enrichment of genes involved in glutathione metabolism, including GPX-1 and GPX-2, which together catalyze the reduction of organic hydroperoxides and H_2_O_2_ by glutathione, protecting cells against oxidative damage (labeled “glutathione metabolism”). Also enriched were glutathione-*S*-transferases (GSTs) of the mu, theta, and omega classes (GST Mu-1, GST Mu-5, GST theta-1, and GST omega-2), involved in detoxification of electrophilic compounds (including carcinogens, therapeutic drugs, environmental toxins, and products of oxidative stress by conjugation with microsomal GST), and microsomal GST 3 involved in the production of leukotrienes and prostaglandin E.

Two other groups enriched in the IMP-1-associated granules include mRNAs that are made in the nucleus and imported into the mitochondrion (41), including the mitochondrial ribosomal components (labeled “mitochondrial ribosome”) and some nuclear-encoded complex I–associated mRNAs of the electron transport chain (labeled “mitochondrial complex I”). Specifically, mRNAs identified in the IMP-1-associated fraction include seven subunits of the nuclear-encoded mitochondrial ribosomal protein small subunit (MRPS) complex (6, 12, 16, 18c, 24, 33, and 36) and five subunits of the mitochondrial ribosomal protein large subunit (MRPL) complex (14, 23, 36, 53, and 57). The gene lists used for these analyses, and the genes included in the Gene Ontology term enrichment, are provided in the supporting information (Table S2 and S4).

### Corresponding mRNAs were pulled through in IMP-1-associated complexes from breast tumor cells

**Figure 8:**
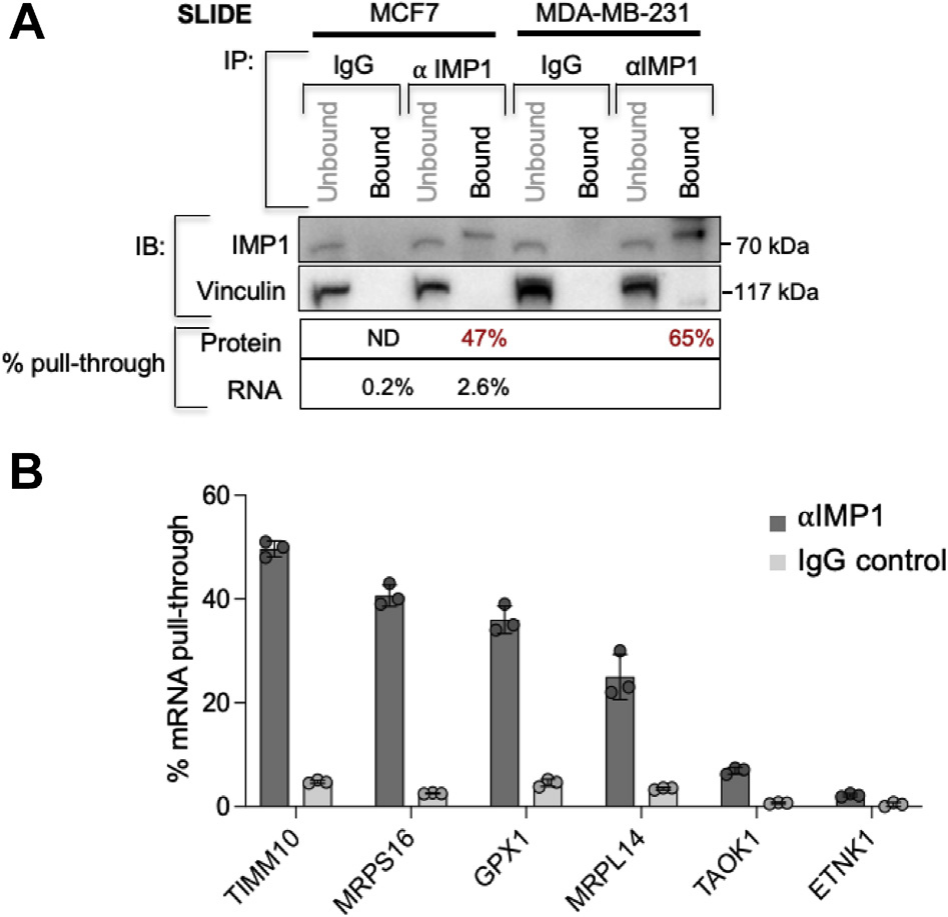
**Confirmation of ROS detoxifiers and mitochondrial mRNAs amongst IMP-1-binding targets in breast cancer cell lines.***A*, comparison of RNA targets enriched by SLIDE processing of IMP-1 RIP from breast cancer cell lines. MCF-7 and MDA-MB-231 human breast cancer cell lysates were incubated with anti-IMP-1 antibody or an IgG control, and the lysate + antibody mixtures were subjected to purification by SLIDE (as per Fig. 4), and the efficiency of pull through of IMP-1 protein and RNA-binding partners was calculated (n = 3). Six mRNAs were assayed by qRT–PCR, including four specifically associated in RIP fractions of mouse mammary epithelial cells, and two excluded from these fractions (TAOK1 and ETNK1; Fig. 6). ETNK1, ethanolamine kinase 1; IgG, immunoglobulin G; IMP-1, insulin-like growth factor 2 mRNA-binding protein-1; qRT, quantitative RT; RIP, RNA immunoprecipitation; ROS, reactive oxygen species; SLIDE, Sliding Lid for Immobilized Droplet Extractions; TAOK1, thousand and one amino acid kinase 1.

As a comparison to these results from mouse breast cells, we tested whether corresponding mRNA-binding species were associated with IMP-1 in human breast tumor cells. Lysates of MCF-7 and MDA-MB-231 cells were purified by anti-IMP-1 SLIDE–RIP, showing a protein purification efficiency of approximately 47% for MCF-7 cells, together with a yield of 2.6% mRNA. This included over sevenfold enrichment (IMP-1–IgG) and >25% total yield of GPX-1 (glutathione metabolism), MRPS16, MRPL14, and translocase of inner mitochondrial membrane 10 (mitochondrial targeted mRNAs), compared with <7% yield of excluded mRNA species (thousand and one amino acid kinase 1 and ethanolamine kinase 1) (Fig. 8).

### Preliminary evaluation of potential functional role of IMP-1-bound mRNAs

**Figure 9:**
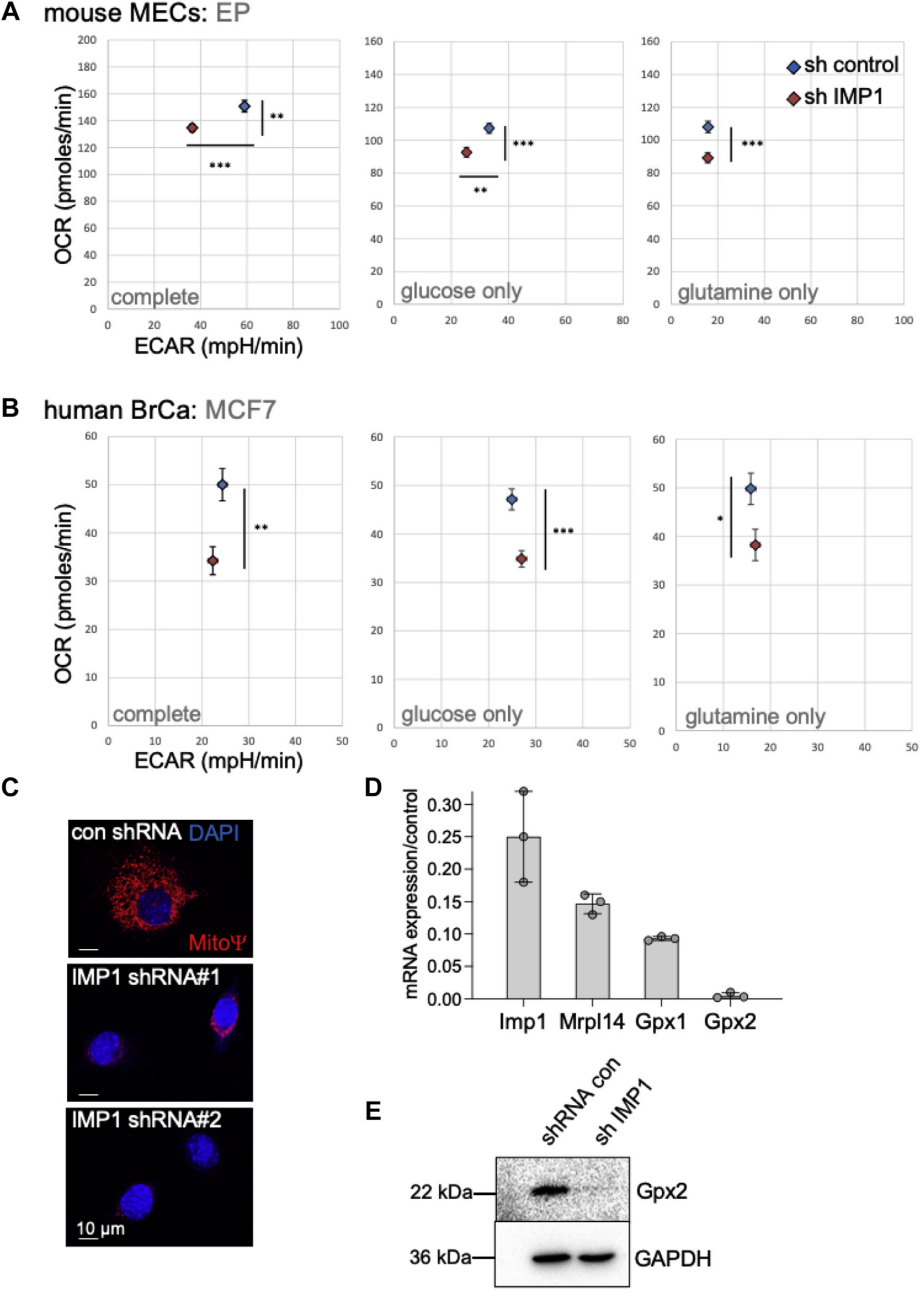
**Functional evaluation of IMP-1-bound mRNAs.***A*–*C*, mitochondrial function is impaired in IMP-1-knockdown cells. Mitochondrial activity was assayed in either mouse EP cells (*A*) or human breast cancer cells (MCF-7 cells; *B*) using Seahorse assay. About 16,500 EP and 12,500 MCF-7 cells were used for each assay shown (representative of three assays). Assays were run either with complete media (with 25 mM glucose and 4 mM glutamine) or with media with only one of these carbon sources, as indicated. ∗∗*p* < 0.05; ∗∗∗*p* < 0.001. *C*, MitoTracker staining of EP cells confirmed a reduced mitochondrial membrane potential (MitoΨ) in IMP1-knockdown cells (shown for two shRNAs). *D* and *E*, glutathione peroxidase (GPX) mRNAs were reduced in IMP1-knockdown cells. Relative (mRNA) was assayed for IMP1-binding mRNA species GPX-1 and GPX-2 in EP cells after (*partial*) knockdown of IMP-1, alongside the mitochondrial ribosomal protein Mrpl14 mRNA, also pulled through in association with IMP-1 (*D*). Western blotting confirmed that GPX-2 protein was also depleted (*E*). ECAR, extracellular acidification rate; EP, epithelial; IMP-1, insulin-like growth factor 2 mRNA-binding protein-1; OCR, oxygen consumption rate.

Given the number of mitochondrial mRNAs bound to IMP-1, we assessed mitochondrial function in IMP-1-knockdown mouse breast EP cells and human breast tumor cells. Oxygen consumption and extracellular acidification (an indicator of lactate efflux and glycemic fraction) were measured by Seahorse assay: this revealed that the consumption of oxygen was significantly reduced in IMP-1-knockdown cells (Fig. 9, *A* and *B*). Lactate is the end product of the cytoplasmic oxidation of glucose, and extracellular acidification rate was thus not affected when glutamine was provided as the carbon source for the tricarboxylic acid cycle for untransformed mouse EP cells. MCF-7 cells show the high glycemic index typical of Warburg-adapted cancer cells; despite major adaptations of mitochondrial function in cancer cells, these mitochondria also require IMP-1 for their activity. Less mitochondrial activity is often correlated with decreased mitochondrial membrane potential, shown by staining with MitoTracker Red (Life Technologies; Fig. 9*C*). We have also shown that IMP-1 binding stabilizes specific GPX mRNA species, namely GPX-1 and GPX-2, reflected at the protein level as an almost complete depletion of GPX-2 protein, and loss of both GPX-1 and GPX-2 mRNAs in EP cells (along with 85% depletion of another mRNA-binding partner [MRPL14]) (Fig. 9, *D* and *E*). GPX-1 and MRPL14 mRNAs are also depleted (>60%) from human breast tumor MCF-7 cells (GPX-2 is not expressed in MCF-7 cells; Fig. 8*B*). This preliminary evaluation illustrates the functional impact of IMP-1 for selected binding mRNAs in both normal and tumor breast cell types.

## Discussion

### The application of ESP to the discovery of RNA-binding partners for proteins

ESP has been shown to be useful for cell, DNA, and protein isolation from complex biological samples (29, 37, 42). The main time-saving and labor-saving aspects of ESP methods are the substitution of the standard and manual pipetting operations with a swift and coordinated wash *via* magnetized bead adsorption. This can have profound impact because increasing the speed of purification can promote the identification of more weakly bound or labile complex components (29). This may particularly apply to RNA–protein interactions because the potential for exchange during the rapid wash procedure applied with ESP technologies is low. Indeed, the potential for exchange of interactors was one of the drivers for development of crosslinking technologies to the study of RBP complexes. These concerns arose from a letter to the editor of *RNA* in 2004, which showed that 90 min of coincubation of a lysate containing an RNA-binding protein (HuR) together with a cell lysate containing a known specific binding partner, the *fos* mRNA species, was more than enough to promote their interaction (8). We showed in a previous publication that an antibody–protein complex designed to mimic lower affinity interactions showed 80% dissociation during a timecourse of 100 min (29). Only 10 min in a moderate salt wash buffer promoted 50% complex dissociation, whereas 1 min showed insignificant losses and, importantly, the wash was still effective. Therefore, the timescale for the ESP purification (in the order of seconds) prevents reassociation artifacts.

### Summary of advantages of ESP over RIP–chromatin immunoprecipitation and CLIP technologies

Techniques for identifying the RNAs that associate with specific RBPs have become increasingly sophisticated (10, 43, 44). However, the downsides of crosslinking of RNA to RBP have been noted before in a publication that showed optimal isolation conditions for a classic “RIP–chromatin immunoprecipitation” (45). A summary of the processing aspects of the ESP-based technologies, IFAST and SLIDE, *versus* one of the examples of CLIP technologies, PAR–CLIP (46, 47, 48, 49), is shown in Figure 1.

We summarize these potential advantages of ESP:

#### Almost quantitative yields

The amount of pull through of any given RBP is related to the avidity of the antibody for the protein of interest (shown by the almost quantitative extraction of a FLAG-tagged version of IMP-1 by the high-avidity anti-FLAG antibody [Fig. 4*B*]). For the anti-IMP-1 antibody, the total yield is approximately 60% of total. Unlike previous RIP studies, we can claim to examine the binding interactions of the majority of RBPs, instead of <1% of total (50). This also offers the opportunity to study endogenous level proteins, whereas RBPs that are overexpressed can be mislocalized (38).

#### Starting amounts of lysate

The amount of starting material required is over 100× less than for a typical CLIP procedure, given there are virtually no losses. Indeed, the sensitivity could be enhanced still further, depending upon the output required. This is especially important for human samples and samples of purified or limiting cells of any source (such as subpopulations of tumor cells or stem cell populations).

#### No mechanistic bias is required

The underlying assumption of crosslinking technologies is that RBPs require direct contact with mRNA-binding partners to affect their stability, delivery, or translation. However, this assumption may not apply to many important RBP interactions that govern supermolecular complex granules (32, 51); the isolation of RNA immunoprecipitates by ESP does not require that the RBPs have direct contact. Thus, although there is value in understanding the precise sites for RNA interaction (*e.g.*, for fragile X mental retardation protein (52)), this is not always necessary.

#### Parallel handling and rapid processing

Prior characterization of the performance of the ESP devices has shown that specific but low-affinity reactions are preserved by rapid pull through of magnetic beads through air or oil. This enhances the typical RIP–chromatin immunoprecipitation protocols by offering an expedited processing procedure, making the starting material less prone to degradation and requiring less handling, which makes loss of key species during wash procedures less likely.

Note that the results of this assay are limited by the nature of the analysis of the complex components; here, the results are restricted to probes that appeared on the microarrays, eliminating important regulatory non-mRNA species such as long noncoding RNA, noncoding RNA, or miRNAs. However, this technique is entirely compatible with other techniques such as RNA-Seq or targeted arrays, which would reveal these species. It is also dependent upon the antibodies used for immunoprecipitation; for example, antibodies previously reported to identify IMP-1–IGF2BP1 partners did not pass our validation criteria (7, 53). Furthermore, mass spectrometry is entirely compatible with ESP–RIP, allowing a full dissection of protein components of RBP particles.

### Comparison of the results of ESP purification of IMP-1-binding RNAs with other studies

IMP-1 has been implicated in a variety of aspects of mRNA metabolism and expression, from the stabilization of mRNAs by blocking miRNA-binding sites (17, 54) to the localization or translation of cognate proteins (55, 56). Functionally, this protein is important to cell survival, cell migration, and chemoresistance (57, 58, 59, 60), and this underlies the focus on determining its mechanism of action.

**Figure 10:**
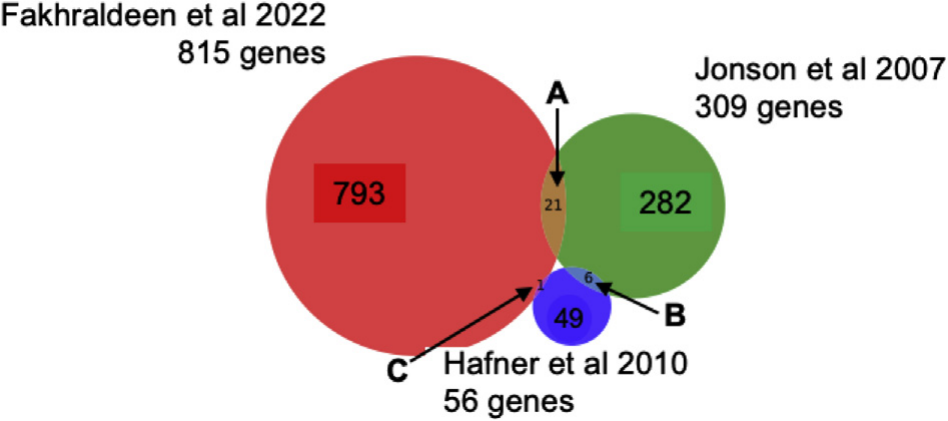
**Comparison of published mRNA-binding partners for IMP-1.** The overlap of mRNA gene lists identified as binding partners for IMP-1 is shown (gene lists provided in Table S3). The general characteristics of the studies are summarized in Table 1. Thus, both studies differ from our study of endogenous IMP-1 in mouse mammary epithelial cells as follows: Jonson *et al.* (38) and Hafner *et al.* (39) overexpressed a FLAG-tagged version of IMP-1 in 293T cells, and Hafner *et al.* analyzed retrieved sequence tags after PAR–CLIP analysis. IMP-1, insulin-like growth factor 2 mRNA-binding protein-1; PAR–CLIP, photoactivatable ribonucleoside–enhanced crosslinking and immunoprecipitation.

There are parallel data for several types of RIP analyses of IMP-1 in different cell types; the results of five studies, including this one, are shown in Table 1 (7, 38, 39, 61). Nielsen *et al.* (38) exogenously expressed FLAG-tagged IMP-1 to determine potential mRNA interactions in human 293T cells; they found that IMP-1 associates with a considerable proportion of the total transcriptome (3%) and that IMP-1 colocalizes with 100 to 300 nm intracellular granules. The study started with 100× more cell lysate and did not report total yields ((38)). Overall, the results showed >300 specific mRNA species associated with IMP-1, which is in the same order of magnitude as our study, where our study relied on the low level of endogenous expression in mouse mammary cells as the immunoprecipitation target. To test for consensus IMP-1 mRNA-binding partners, we compared the gene lists generated by Jonson *et al.* with this study and found only 21 genes in common (Fig. 10 and Table S3), despite the fact that the majority of target mRNAs were expressed in both 293T and mouse mammary EP cells. However, we sampled 10 of the mRNA partners identified by Jonson *et al.* in SLIDE-purified immunoprecipitates of endogenous IMP-1 from human 293T cells and confirmed them to be highly enriched (Fig. 5*C*). In addition, the mRNAs overlapping between these studies included representatives of the functional cohorts identified in this study (discussed later): These are SH3BGRL3, one of a relatively uncharacterized family of three thioredoxin-like proteins, and two nuclear-encoded mitochondrial proteins, translocase of inner mitochondrial membrane 17B, an integral mitochondrial membrane protein in the translocase of inner mitochondrial membrane 23 complex, and NME/NM23 nucleoside diphosphate kinase 4, a nucleoside disphosphate kinase that binds predominantly to the complex mitochondrial lipid, cardiolipin (Table S3).

**Table 1:** Comparison of RNA associations for CRD-BP defined by different RIP techniques

Study	Method	Scale/cell type	IMP-1 target	RNA crosslink/RNA labeling	Assay	Proportion of IMP-1 retrieved for analysis; number of RNA-binding partners
Jonson *et al.*, 2007 (38)	RIP	1.20 × 10^8^ 293T cells	FLAG-tagged Exogenous	None None	Affymetrix U133 Plus 2.0 array	ND 3% total mRNA in multicomplex granules; 352 mRNAs (>3× enriched, *p* < 0.05)
Hafner *et al.*, 2010 (5)	PAR–CLIP	>10^8^ 293T cells	FLAG/HA-tagged Exogenous	UV XLink Labeling *in vivo* with 4SU; labelled *in vitro* with^32^P	Solexa sequencing of cDNA library; ID of interacting sequences	ND 56 mRNAs (+19 mRNAs encoded by mitochondrial genome)
Barnes *et al.*, 2015 (61)	RIP	≈10^6^ HeLa cells	FLAG/HA-tagged mouse sequence Exogenous	None None	Site-directed mutagenesis of IMP-1; focus on three binding partners, CD44, c-myc, and β-actin	ND Not known
Conway *et al.*, 2016 (7) Lambert *et al.*, 2014 (62)	eCLIP	2 × 10^7^ hESC cells	Endogenous FLAG-tagged Exogenous	UV XLink Labeling *in vivo* with 4SU; labeled *in vitro* with^32^P	Comparison of eCLIP and RBNS hits	ND Not known
This study	RIP with ESP	<10^6^ 293T or mammary epithelial cells	Endogenous FLAG-tagged Exogenous	None	Nimblegen array	50–90% IGF2BP1 protein 900 RNA species enriched (*p* < 0.05)

The experimental conditions are listed that relate to approach, cell background, and outcomes of each technique.

Abbreviations: CRD-BP, coding region determinant-binding protein; HA, hemagglutinin; ND, not determined.

Further comparison of both these RIP studies with a gene list generated from retrieved sequence tags after PAR–CLIP from 293T cells (39) showed even less consensus; of the 56 genes retrieved by Hafner *et al.*, there was minimal overlap, even with the RIP study of 293T cells using FLAG-tagged IMP-1 (Fig. 10). CLIP crosslinking techniques include sophisticated statistical arguments to identify significant rates of association of specific RNA sequences. Studies of other cell types (HeLa and human embryonic stem cell) using enhanced CLIP or RIP also showed little or no consensus of IMP-1 mRNA-binding partners (7, 61, 62). The catalog of mRNAs identified in RIP isolates of human embryonic stem cells was not specified (7), but the authors state that there was no overlap between the enhanced CLIP gene set and the genes identified by the RNA Bind-n-Seq technique described by Lambert *et al.* (62) or between those gene lists and the mRNAs destabilized by knockdown of IMP-1. Although IMP-1 was originally isolated as the protein stabilizing c-MYC mRNA in K562 cells (16, 18, 54), this association is not necessarily typical of other cell types, despite widespread expression of c-MYC. Only Barnes *et al.* (61) retrieved the “signature” binding partners c-MYC or IGF2 mRNAs from their RIP study of IMP-1-binding mRNAs in HeLa cells.

Clearly, the reasons for the lack of overlap between the parallel techniques employed by these studies are not yet understood and could be important to resolve. Besides the technical differences inherent in determining significant mRNA interactions, there could be biological differences in binding partners that reflect specific covalent modification of mRNA cohorts that regulate cell phenotypes. For example, target mRNAs such as MYC have been shown to bind IGF2BPs only when modified by N6-methyladenosine (63). This RNA methylation activity is highly regulated during development, differentiation, and carcinogenesis (64, 65).

We are intrigued by the enrichment of mRNAs from the glutathione metabolism pathway (30, 66) and for nuclear-encoded mRNAs encoding mitochondrial proteins (67) in the RIP fractions of mammary EP cells. In general, there is a well-established partnership between regulated RBP activity and mitochondrial mRNA translation and import (41). Mitochondrial function is in turn an important determinant of clonogenic survival and establishment, also associated with IMP-1 activity (68, 69). For example, clustered mitochondria homolog, together with other RBPs, has been shown to regulate the import and expression of a mitochondrial protein network that becomes important under conditions of nutrient deprivation (70, 71). Our data indicate that IMP-1 is required for the mitochondrial activity in both mouse mammary EP cells and human breast cancer cells, thus corroborating the identification of many of the mitochondrial ribosomal and complex I component mRNAs as IMP-1 partners. This functional connection has also been demonstrated by a screen of modifiers of mitochondrial ribosomal translation, which identified predictable ribosomal proteins, along with IMP-1 and other IGF2BP family members (32). One of those family members, IGF2BP2, was previously shown to have an important role in maintaining oxidative phosphorylation and clonogenicity in glioblastoma cells (31).

Another enriched ontology group we identified in this study included genes involved in glutathione metabolism. Preliminary evaluation of this connection showed that the mRNAs for GPX-2 (and GPX-1) were destabilized when IMP-1 was absent. GPX-2 is an intriguing antioxidant particularly important for keeping cells safe in response to natural oxidizers (such as oxidative lipids) and for cancer cells, as protection against oxidative stressors, including chemotherapies (30, 72). This suggests a molecular basis for the implication of IMP-1 and its close paralog, IGF2BP3, in the drug resistance of tumor cells (60, 73, 74, 75, 76, 77, 78).

We have demonstrated the application of a simple enhanced RIP procedure that enables parallel processing and highly efficient retrieval of specific RBP-associated mRNAs. This procedure can be applied to limiting amounts of experimental materials. This technique increases the currently available technologies that can be applied to this field of research and offers the opportunity to evaluate alterations of RBP-associated species under numerous experimental conditions that is not feasible by other means. Our preliminary analysis of the RBP, IMP-1, in cultured mouse mammary EP cells reveals intriguing links to functional components of cancer cell metabolism.

## Experimental procedures

### Cell culture and transfections

MEFs, the EP, and EN substrains of the mouse mammary EP cell line HC11, 293T, MCF-7, and MDA-MB-231 cells were cultured as previously described (19). A construct expressing FLAG-tagged IMP-1 was described in a previous publication (79). Transfections were carried out using Lipofectamine LTX with plus reagent (Life Technologies) according to the manufacturer's instructions.

### RIP

Cells were cultured in 10 cm dishes and lysed in polysome lysis buffer (10 mM Hepes [pH 7.0], 5 mM MgCl_2_, 100 mM KCl, and 0.5% NP-40) with freshly added DTT (1 mM), RNase inhibitor (2 U/μl; catalog no.: 10777019; Thermo Fisher Scientific), and Halt protease/phosphatase inhibitors (as directed; catalog no.: 78442; Thermo Fisher Scientific). Approximately 10^6^ 293T cells (a single 10 cm dish at ∼80–90% confluence) yields 500 μl of lysate, with approximate protein concentration of 4 μg/μl; RIP reactions were processed in batches of 200 μl. For other cell types, such as the breast EP cells described in these studies, 2 × 10 cm dishes were processed into 500 μl of lysis buffer. Lysates were sonicated (10 pulses at 4–5 W) and cleared by spinning 3× at 12,000 rpm for 30 min at 4 ^°^C. Protein concentrations for the whole cell lysates and the unbound fractions were determined using Bradford reagent (Sigma–Aldrich). For immunoprecipitation, approximately 1.2 μg of specific primary antibody (or matched IgG control) were added, together with 5 μl of washed protein G-bound paramagnetic Dynabeads (catalog no.: 10003D; Life Technologies). Antibody binding of RBP complexes was allowed to proceed at 4 ^°^C for the times indicated, before purification using IFAST, SLIDE based, or standard immunoprecipitation. The following antibodies were used: anti-IMP-1 antibody (catalog no.: 8482; Cell Signaling; research resource identifier [RRID]: AB_11179079), anti-FLAG antibody (Sigma–Aldrich; catalog no.: F3165; RRID: AB_259529), or nonimmune rabbit IgG control (Jackson ImmunoResearch; catalog no.: 011-000-003; RRID: AB_2337118). Following the RIP procedures, aliquots from the bound and unbound fractions were harvested and assayed for IMP-1 using Western blotting (to assess efficiency of extraction), and the remaining sample was used for RNA analysis.

### Western blotting

Lysates were analyzed by SDS-PAGE followed by transfer to polyvinylidene difluoride membranes, as described (19). Primary antibodies: anti-IMP-1 (catalog no.: 8482; Cell Signaling; RRID: AB_11179079), diluted 1:1000; antivinculin (catalog no.: 05-386; Millipore; RRID: AB_309711) diluted 1:5000; and anti-GAPDH (catalog no.: 2118; Cell Signaling; RRID: AB_561053) diluted 1:3000 to 1:5000. Secondary antibodies: horseradish peroxidase (HRP) antimouse (catalog no.: 715-035-151; Jackson ImmunoResearch; RRID: AB_2340771); HRP antirabbit (catalog no.: G-21234; Invitrogen; RRID: AB_2536530) diluted 1:5000, or HRP mouse antirabbit IgG (conformation specific) (catalog no.: L27A9; Cell Signaling; RRID: AB_10892860). All antibodies were diluted in 5% milk in Tris-buffered saline–Tween.

### RNA isolation and analysis

Total RNA was isolated using the RNeasy Mini Kit (Qiagen). RNA concentrations and quality were determined using a NanoDrop instrument (Thermo Fisher Scientific; average 260/280 ratio of ∼ 2.1). Reverse transcription was performed as previously described, and specific mRNAs were quantified using qRT–PCR (80). Primer sequences are appended in Table S1.

### Microarray analysis

A Nimblegen 12-plex whole mouse genome microarray chip (Build 100718_MM9_EXP_HX12; comprising 44171 probes, equivalent to 24205 individual genes) was used to assay the relative abundance of each mRNA in complementary DNA (cDNA) libraries made from each sample. RNAs were processed for this analysis according to the manufacturer's instructions; briefly, first strand followed by second-strand cDNA synthesis was performed, followed by RNase cleanup and cDNA precipitation. Double-stranded cDNA (4 μg) was Cy3-labeled and hybridized to microarray chips, which were then washed and scanned. For any given experimental condition, duplicate sets of four samples of cDNA were prepared for analysis by microarray: unbound and bound RNA from anti-IMP-1 immunoprecipitates and anti-IgG immunoprecipitates (specificity control) for analysis. The data were analyzed using Multi-Experiment Viewer freely available software (https://mev.tm4.org).

### Bioinformatic analysis

Raw intensity readings from the microarray analysis were log2 transformed and median centered. These lists were rank ordered, and the rank of genes determined for the mRNAs copurifying with the PMPs, for comparison with the ranking of genes in the unbound fractions. Statistical analysis of independent replicates was used to reflect the significance of fold changes, *p* < 0.01. Enriched gene sets for RNAs pulled through by the specific antibody, anti-IMP-1, were compared with the nonspecific control, IgG fraction. The relative enrichment of RNA species in bound fractions was confirmed independently by qRT–PCR. These confirmed mRNA species, accumulating in anti-IMP-1 immunoprecipitation reactions, were imported into pattern prediction algorithms, such as Search Tool for the Retrieval of Interacting Genes/Proteins (string-db.org) and our own Venn diagram chart making code. Table S2 includes the full list of mRNAs found by our study (IFAST of mouse mammary EP cells) in control IgG RNA immunoprecipitates (IgG RIP, shown ranked for *p* values and fold enrichment), the gene list generated by anti-IMP-1 RIP, and a list of 900 IMP-1 mRNAs bound only in the anti-IMP-1 fraction and not the anti-IgG (*p* < 0.01). Gene lists of mRNAs pulled through by our study and others (38, 39) are included in Table S3. Duplicate and incorrect entries were deleted from gene lists to produce the “curated” versions, and the overlapping gene names are shown in lists D–F, which match the Venn diagram in Figure 10.

### Functional assay of mitochondria

Functional mitochondrial assays were optimized and performed according to the manufacturer's instructions. Briefly, cells were pre-equilibrated with the media indicated (Dulbecco's modified Eagle's medium with 25 mM glucose and 2 mM l-glutamine [complete] or Dulbecco's modified Eagle's medium supplemented with individual substrates at the same concentration as complete media) and then transferred to the Agilent Seahorse Analyzer XFe96 for assay of oxygen consumption rate and extracellular acidification rate. To assess mitochondrial membrane potential (MitoΨ), cells were stained with MitoTracker Red (catalog no.: M7512) according to the manufacturer's instructions.

## Data availability

Data are contained in the article; gene lists identified in RNA immunoprecipitates by this study and others are listed in Tables S2 and S3.

## Supporting information

This article contains supporting information: Table S1 contains primer sequences for qRT–PCR; Table S2 contains detailed gene lists described for the IFAST-derived IMP-1 partners; and Table S3 contains a comparison of three studies of IMP-1 partner mRNAs, seeking overlap.

## Conflict of interest

S. M. B. holds equity in and is employed by Salus Discovery LLC, which has licensed technology described in this article. D. J. B. holds equity in BellBrook Labs LLC, Tasso Inc, Stacks to the Future LLC, Lynx Biosciences LLC, Onexion Biosystems LLC, and Salus Discovery LLC. All the other authors declare that they have no conflicts of interest with the contents of this article.
