# Supplementary material for: Enhanced immunoprecipitation techniques for the identification of RNA-binding protein partners: IGF2BP1 interactions in mammary epithelial cells
Source: J Biol Chem. 2022 Jan 29;298(3):101649. doi: 10.1016/j.jbc.2022.101649 (PMC8891971; doi:10.1016/j.jbc.2022.101649)
Supplement: Supplemental Table S1 [file mmc1.docx]

**Table S1. Primers used for qRT-PCR**

| Species/Gene/Accession # | Forward primer (5’-3’) | Reverse primer (5’-3’) |
| --- | --- | --- |
| mCoxIV (NM_009941) | GGAAGACAGTTGTGGGCAT | CACCCAGTCACGATCGAAAG |
| hCoxIV (NM_001861) | TCGGTTTCACCGCGCTCGTTAT | TGTCCAGCATCCTCTTGGTCTG |
| mTimm10 (NM_013899) | TGCGTGCCTCCCCACTACAAG | ATCCTCTCATGGATGTCCAAGTAC |
| hTimm10 (NM_012456) | TGTGTGCCTCCTCACTACAAGG | ATCCGCTCATGGATGTCCAGGT |
| mMrpl14 (NM_026732) | GAAAGCACTCATCGTGGGACAC | GAAGGCTGGTCGGGATAGGTAT |
| hMrpl14 (NM_032111) | GTGCGATTCAGAAGATGACGCG | GTAGTATCTGGTCGCCCACCTT |
| mMrps16 (NM_025440) | CTCACCTCTCTAAGCCTATGGAG | TCTGAGACGCTAAGAGGACCTC |
| hMrps16 (NM_016065) | GTTGCCCTCAACCTAGACAGGA | CCGTTTCCTTCGCAGTCTCTCA |
| mMrpl24 (NM_026591) | CCATCTCTGATGAAGACTGGCAC | AGTTTCGCTGCCGAACGACTTG |
| mMrpl41 (NM_001031808) | CTCTTCCAGGAAACAGTTGCACC | GAACAGCTTGCCTTCCTGTGTG |
| mFcf1 (NM_028632) | CAATGATGGACTGTCTGTATGCC | CAATCGGTCAAACCGTGGATCC |
| mGpx1 (NM_008160) | CGCTCTTTACCTTCCTGCGGAA | AGTTCCAGGCAATGTCGTTGCG |
| hGpx1 (NM_000581) | GTGCTCGGCTTCCCGTGCAAC | CTCGAAGAGCATGAAGTTGGGC |
| mRps27 (NM_027015) | CCTACTTTATGGACGTGAAATGCC | CTGTCAGCCTTGCTTTTCCACC |
| mGpx1 (NM_008160) | CAGGAGAATGGCAAGAATGA | GAAGGTAAAGAGCGGGTGAG |
| mGpx2 (NM_030677) | CCAGGAATCTCCCACTGTTT | GGGACGATATTCAGGGAATG |
| mGstm1 (NM_010358) | CCTGCCCACGTTTCTCTAGT | GGTGCTGTGGTCTTCTCAAA |
| mGstm5 (NM_010360) | TTTGGAGAAGA TTGCTGCAT | CTCTGGCTCAGCATAAGCAC |
| hGstm5 (NM_000851) | CACATGGAGCTGGTCAGACTGT | CTTGTCTCCTGCAAACCATGGC |
| mPsmb4 (NM_008945) | TGAAGAGCTGTTGGGAGATG | GTCAGCATAGCCTCCAATGA |
| mSdhb (NM_023374) | AGAAGGCATCTGTGGCTCTT | GAGACTTTGCTGAGGTCCGT |
| mCox6c (NM_053071) | ATTGGCTACCATGAGTTCCG | AGCAATATGAACCCGCAGA |
| mRps27l (NM_026467) | TGTGAAA TGTCCAGGTTGCT | GCTGGCACAACACAGTTGA |
| mUqcrq (NM_025352) | CACGCGTCTATCTTCTGTCC | CTGAAATAGCTTGGGAAGGC |
| mSorbs2 (NM_172752) | GCTGGAGGAAGAAACTCAGG | GGCACCAGGGTGTTAGACTT |
| mRnf150 (NM_177378) | GTATCAGAGGCAATCAGGCA | CTGTCCCAGAACTTGCTTCA |
| mSlc16a4 (NM_146136) | CATTTCTGGATGGATTGCTG | GCCATAAGGAGTGGGAATGT |
| mGpatch8 (NM_001159492) | GACTGCACCAGAAGTCTCCA | GGAGGAACGATGTGAGGAAT |
| mEtnk1 (NM_029250) | CTTACCCATGGTGGTCCTTT | CAATTCCAATCGGATAGGCT |
| hEtnk1 (NM_018638) | CCACAGGATTTGCAGATGAAGAC | TGGCAAAGCACAACAGGTGAGC |
| mZc3h18 (NM_001029993) | CTACAGCTCCTATTCTAGCCGC | CCTTGGTTCTGACTGGAGGTCT |
| mRock2 (NM_009072) | AACTGTGATCCCAAGGGAAG | CAGCAGCAGTATGCCATCTT |
| mDtnb1 (NM_007886) | CTACGGAGCAGGAGAGAACC | TCTTTCACAGCCGAACTGTC |
| mEea1 (NM_001001932) | TCAAGGCAGAGTGGACTCAC | TTCCTCCTTCTTCACCTGCT |
| mCenpf (NM_001081363) | TAGGAATGGCGTCCTCTTCT | ATTTGCAGCAACAAACAAGC |
| mTaok1 (NM_144825) | AGGAACTGGAGCTGCTGAA T | GACCCTCTGTTCCAGCTCTC |
| hTaok1 (NM_025142) | GGCGTCATAACTTAGAGCAGGAC | TTGTGTTGAGGTGGCGGAACTC |
| mLcor (NM_172154) | AGCACAAAGAACCAAAGCCT | CCAGAGGTGAGTCTTGGTCA |
| mHuwe1 (NM_021523) | TATCCGTCGGGAAGTCCTAC | AGCTATTTGAGGAGGGAGCA |
| mChka1 (NM_013490) | CTGAAGGGCAAGTGAACAGA | ACCTGCATACCCACAGTCAA |
