# Supplementary material for: Enhanced immunoprecipitation techniques for the identification of RNA-binding protein partners: IGF2BP1 interactions in mammary epithelial cells
Source: J Biol Chem. 2022 Jan 29;298(3):101649. doi: 10.1016/j.jbc.2022.101649 (PMC8891971; doi:10.1016/j.jbc.2022.101649)
Supplement: Supplemental Table S4 [file mmc4.docx]

**STRING Gene Ontology analysis**

**Anti-IMP1** (815 genes)

mmu00480 Glutathione metabolism 11/69 genes FDR 0.0172

Gstt1,Gstm5,Gstm1,Mgst3,Gstk1,Gsto2,Ggt5,Gpx1,Gpx2,Ggct,Cml1

GOCC:0005761 Mitochondrial ribosome 15/84 FDR 0.00020

Mrpl52,Mrps18c,Mrpl36,Mrpl57,Mrpl14,Mrps33,Mrps6,Ndufa7,Mrpl23,Mrps34,Mrps16,Mrps12,Mrps21,Mrpl24,Mrps14

GO:0005747 Mitochondrial respiratory chain complex 1 8/47 genes FDR 0.0183 Ndufa11,Ndufa2,Ndufa1,Ndufb3,Tmem261,Ndufc2,Ndufa7,Ndufb5

**Anti-IgG** (control; 1170 genes)

CL:543 TATA box binding protein associated factor (TAF), and C-terminus of histone H2A 14/25 FDR 6.63e-05

Hist1h2ab,Hist2h2aa2,Hist1h4j,Hist1h3c,Hist2h3b,Hist1h4b,Hist1h4c,Hist1h4d,Hist1h2ae,Hist1h4h,Hist1h4i,Hist1h4k,Hist1h3e,Hist2h3c2
